# Seed size and capitulum position drive germination and dormancy responses to projected warming for the threatened dune endemic *Cirsium pitcheri* (Asteraceae)

**DOI:** 10.1002/ece3.7109

**Published:** 2020-12-21

**Authors:** Finote Gijsman, Pati Vitt

**Affiliations:** ^1^ Department of Biology Northwestern University Evanston IL USA; ^2^ Chicago Botanic Garden Glencoe IL USA; ^3^ Present address: Lake County Forest Preserve District Libertyville IL USA

**Keywords:** *Cirsium pitcheri*, climate change, dunes, survival curves, time‐to‐event analyses

## Abstract

Among coastal plant species at risk from rapid environmental changes is the North American Great Lakes dune endemic *Cirsium pitcheri*. Despite being listed as federally threatened, little is known about how *C. pitcheri* seed attributes influence germination and dormancy‐break patterns in the context of climate change. Following a previous work where we found differences in the number and weight of *C. pitcheri* seeds among capitulum positions and study sites, here we examine the effects of seed attributes (capitulum position, seed weight, and site of origin) on the proportion and timing of *C. pitcheri* seed germination under temperature treatments that simulate projected warming in the Great Lakes (20/10, 25/10, and 30/10°C day/night). Our results demonstrate that *C. pitcheri* produces diverse cohorts of seeds with seed attributes that significantly influence the timing and probability of germination over a 3‐year soil seed bank. *Cirsium pitcheri* seed germination proportions were highest at 20°C and decreased successively at 25 and 30°C. Seeds from terminal capitula also had higher germination proportions and took longer to germinate than those from secondary capitula. Lastly, the effect of seed weight on germination probability depended on site of origin and capitulum position, with all effects varying in size and significance over time. Ultimately, our results highlight the considerable differences in germination patterns exhibited by seeds from different capitulum positions and sites of origin and provide insight into the dormancy‐break patterns that *C. pitcheri* might experience under predicted temperature rise in the Great Lakes region of North America.

## Graphical abstract

*Cirsium pitcheri* is a threatened thistle species endemic to the dunes of the North American Great Lakes. The attributes of seeds that it produces, like seed weight and site of origin, significantly influence seed germination and dormancy responses to predicted warming.

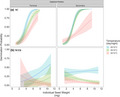

## 1 INTRODUCTION

Coastal dune ecosystems are among the most fragile and threatened environments across the world (Emery & Rudgers, 2010; Frosini et al., 2012; Martínez & Psuty, 2004). Anthropogenic activities such as shoreline development, tourism, and pollution have significantly impacted dune structure, stability, and ecosystem functioning (Brown & McLachlan, 2002; Frosini et al., 2012; Martínez & Psuty, 2004), and have increased erosion and habitat fragmentation (Frey et al., 2016). Plant species with narrow dune habitat specificities are especially vulnerable to these disturbances as they affect the establishment and survival of individuals in changing dune landscapes (Martínez & Psuty, 2004).

Habitat and other environmental changes induced by climate change are expected to further exacerbate dune plant population declines (Frosini et al., 2012; Seabloom et al., 2013; Staehlin & Fant, 2015). While plants growing in dune ecosystems are adapted to withstand environmental stresses like resource limitation, low moisture, high temperatures, and periodic burial (Brown & McLachlan, 2002; Hamzé & Jolls, 2000; Martínez & Psuty, 2004), the increased intensity and frequency of natural and anthropogenic ecosystem perturbances predicted by climate models may significantly reduce the appropriate climate envelopes for dune plant species (Frosini et al., 2012; Seabloom et al., 2013). Changes in abiotic factors like precipitation and temperature will also likely affect plant developmental responses and impact life stage performances of subsequent plant generations (Gray & Brady, 2016; Walter et al., 2016).

Among at‐risk dune species with narrow habitat specificities is the Great Lakes, North American dune endemic *Cirsium pitcheri* (Torr. ex Eaton) Torr. & A. Gray (Asteraceae, tribe Cynareae). As a monocarpic perennial species, *C. pitcheri* flowers and sets seeds only once in its lifetime after growing as a rosette for 4–8 years (Havens et al., 2012; Jolls et al., 2019; Loveless, 1984). Flowering in this species is determinate, beginning with terminal capitula at the apex of main stems and progresses basipetally to axillary, secondary, and tertiary capitula that are produced at the tips of first‐ and second‐order branches, respectively (Gijsman et al., 2020; USFWS, 2002). At the time of dispersal in late July, *C. pitcheri* seeds are dormant and are attached to a long pappus that helps parachute them across the dune (Chen & Maun, 1999; Hamzé & Jolls, 2000; Keddy & Keddy, 1984; USFWS, 2002). Once dispersed, *C. pitcheri* seeds are subjected to multiple stressors in the infertile dune sand substrate including low moisture, high sand temperatures, and burial (Hamzé & Jolls, 2000). With an ephemeral between‐year soil seed bank, a combination of sand burial and multiple episodes of low temperature (cold) stratification is required to break seed dormancy (Chen & Maun, 1999; Hamzé & Jolls, 2000). Studies investigating *C. pitcheri* seed germination have demonstrated that seed attributes like mass and source influence seed dormancy (Hamzé & Jolls, 2000), as well as seedling emergence (Staehlin & Fant, 2015) and survival in dune environments (Chen & Maun, 1999).

The successful production of viable seeds for *C. pitcheri* has even greater import given its threatened status (COSEWIC, 2000). Population viability models for *C. pitcheri* predict its extinction time to be <20 years (Havens et al., 2012; Jolls et al., 2015). Factors like shoreline development (USFWS, 2002), lake level fluctuations (Staehlin & Fant, 2015), and seed predation by the non‐native weevil, *Larinus planus* (Fabricius, 1792) (Havens et al., 2012; Louda & O'Brien, 2002) are expected to contribute to *C. pitcheri* population declines across its range. Further population declines are also expected due to climate change as temperatures in the Great Lakes region rise (Hayhoe et al., 2010) and early life stages, like seed germination and seedling emergence, are impacted (Staehlin & Fant, 2015). However, despite the bleak predictions for *C. pitcheri* population persistence, little is known about the impact of climate change on *C. pitcheri* seed dormancy‐break patterns and how seed attributes affect the timing and probability of seed germination in the context of climate change.

Following previous work where we found differences in the number and weight of *C. pitcheri* seeds produced in terminal and secondary capitula between two study sites (Gijsman et al., 2020), we conducted an experiment that examined the effect of seed attributes (capitulum position, site of origin and seed weight) on *C. pitcheri* seed germination in the context of climate change. We first broadly assessed the effect of seed attributes on cumulative *C. pitcheri* seed germination at three temperature treatments that simulate projected temperature rise in the Great Lakes and then constructed a more fine‐grained distribution of germination times and probabilities by tracking the timing and germination of *individual* seeds. Altogether, this study aims to improve current knowledge on *C. pitcheri* germination ecology and better predict its population dynamics and responses to climate change.

## 2 MATERIALS AND METHODS

### 2.1 Seed collection and sample sizes

Seeds used in this study were collected in June 2016 and 2017 from a randomized sample of *C. pitcheri* flowering plants at two sites in Door County, Wisconsin: The Ship Canal Nature Preserve (SC, *N* = 34 plants in 2016, 15 in 2017) and Whitefish Dunes State Park (WFD, *N* = 43 plants in 2016, 15 in 2017). We used seeds collected from senescing flowering plants in 2016 for germination trials tracking cumulative seed germination (*N* = 1,217 at SC, 1,078 at WFD) and in 2017 for trials tracking individual seed germination (*N* = 1,073 at SC, 1,277 at WFD) (see Gijsman et al., 2020 for collection methods). For each site and year, we haphazardly selected a subsample of the total number of viable seeds produced by flowering plants in terminal and secondary capitula. However, because flowering plants across sites and years produced different numbers of capitula, sample sizes for seeds from each site, capitulum position, and year vary slightly (see Table S1 for specific sample sizes and experiment replicate numbers).

### 2.2 Seed preparation and germination trials

Prior to our germination trials, we surface‐sterilized seeds through submersion in a 0.25% sodium hypochlorite (bleach) solution for 1 min and rinsed seeds with distilled water to ensure the removal of contaminating microbes. Any excess moisture from the surface of the seeds was removed using lint‐free Kim wipes. For germination trials tracking cumulative seed germination, we plated a randomized subsample of 20 seeds from a given capitulum position and site of origin onto Petri dishes lined with dampened, sterilized germination paper, and randomly assigned Petri dishes to one of three temperature treatments (20/10, 25/10, and 30/10°C day/night, hereafter “20, 25, and 30°C”). These temperatures fall within ranges previously tested for *C. pitcheri* (Chen & Maun, 1998, 1999; Staehlin & Fant, 2015) and simulate predicted temperature scenarios that are expected to influence its range (Hayhoe et al., 2010; Vitt et al., 2010).

To investigate the timing and probability of germination for individual *C. pitcheri* seeds, we weighed seeds to the nearest 10^−4^ g using a Sartorius Mettler Toledo balance prior to surface sterilization and tracked seed attributes (site of origin, capitulum position, seed weight) of individual seeds using 96‐well plates. We randomly assigned each seed to a temperature treatment and plated seeds onto Petri dishes lined with two layers of differently colored germination paper, to facilitate seed tracking and visualization of individual seed germination. We hole‐punched the top layer of germination paper, labeled holes with a seed‐tracking letter and plated up to 15 seeds for a given capitulum position and site of origin.

Once prepared, we placed Petri dishes into incubators set to 3°C for a cold stratification period of 8 weeks, to break seed dormancy by simulating natural winter conditions *C. pitcheri* seeds must overcome to germinate (Chen & Maun, 1998). However, despite surface sterilization with bleach and the use of sterile equipment, fungal growth was a significant problem in our experiment, particularly for seeds from WFD. To control the spread of fungus, we treated Petri dishes with a dilute solution of captan fungicide (3.5 g/L), a tissue culture and seed sterilization treatment (Falloon, 1987; Payamnour et al., 2014).

After the 8‐week cold stratification period, we placed Petri dishes into light‐controlled incubators, alternating 12/12 day/night hours at 20/10, 25/10, and 30/10°C. We tracked seed germination, defined as radicle emergence >1 mm, every 2 days for up to 10 weeks. Once germinated, we transferred seeds to labeled starter trays with a soil mix composed of two‐parts potting mix and one‐part sand and grew seedlings in their respective germination temperatures until large enough to be transplanted to sandboxes at the Chicago Botanic Garden (CBG) for use in future dune restoration projects.

As *C. pitcheri* seeds are viable for up to 3 years in the soil seed bank and require multiple consecutive periods of cold stratification to fully break dormancy (COSEWIC, 2000), we placed any remaining ungerminated seeds back into cold stratification at 3°C for an additional 8 weeks, followed by a subsequent warm‐temperature germination period of 10 weeks at their previously assigned temperature. We subjected all ungerminated seeds to up to three cold stratification‐germination rounds to simulate an approximate 3‐year seed bank. Any seeds that germinated within a 3°C cold stratification period were included in germination proportions within each round of cold stratification‐germination. For germination trials tracking individual seed germination, we recorded the following additional time‐to‐event data over the course of each cold stratification‐germination round: binomial germination success (y/n), germination within cold stratification round (y/n), number of germination and cold stratification rounds experienced prior to germination, and the number of days taken to germinate from the start of the experiment (day 0 = first day of first cold stratification round).

### 2.3 Statistical analyses

We used generalized linear models (GLMs) with a binomial distribution to test for the effects of site of origin, temperature, capitulum position, and their interactions on cumulative *C. pitcheri* germination in each cold stratification‐germination round. For each cold stratification‐germination round, we selected minimal adequate models that best described our data using a stepwise, backward elimination approach that sequentially tested the removal of a seed attribute predictor term or interaction with a threshold of 5% (*p* ≤ .05) for rejecting simpler models (Crawley, 2015).

For individual seed germination trials, we used GLMs with a binomial distribution to test for the effects of site of origin, capitulum position, temperature, seed weight, and their interactions on total *C. pitcheri* seed germination proportions at the end of all cold stratification‐germination rounds. We also tested for differences in the mean time to seed germination among seed attribute predictors using a GLM with a negative binomial distribution due to overdispersion with Poisson models.

To investigate the rate of *C. pitcheri* seed germination over time, we used nonparametric Kaplan–Meier (K‐M) time‐to‐event analyses which assume no survival curve shape and account for censoring in our data. Seeds that did not germinate by the end of the experiment were treated as censored data and given a censoring time equivalent to the final day of the experiment (day 375), when germination was last checked. For each capitulum position, we made pairwise comparisons of the survival curves of seeds from different site‐temperature combinations using log‐rank tests and plotted reverse Kaplan–Meier curves (1 − K‐M survival probability) that show cumulative germination proportions over time. To obtain estimates for the effects of seed attributes (site of origin, capitulum position, and temperature) on seed germination survival curves, we ran a series of multivariate accelerated failure time (AFT) models comparing the hazard ratios of seeds within each cold stratification‐germination round. Hazard ratios describe estimates of event probabilities, in this case germination, within a treatment group in comparison with a control group. A value greater than one represents an increase in the probability of an event taking place within a treatment group compared to a reference, less than one a reduction and equal to one represents no difference. We conducted our comparisons of hazard ratio estimates within levels of seed attribute predictors, using the site of origin level “SC,” capitulum level “terminal capitula” and temperature level “20°C” as references. We conducted all analyses in R (R Core Team, 2016) and used the survival (Therneau, 2015) and survminer (Kassambara et al., 2019) packages for time‐to‐event analyses and ggplot2 package to graph results (Wickham, 2016).

## 3 RESULTS

### 3.1 Cumulative seed germination

**FIGURE 1:**
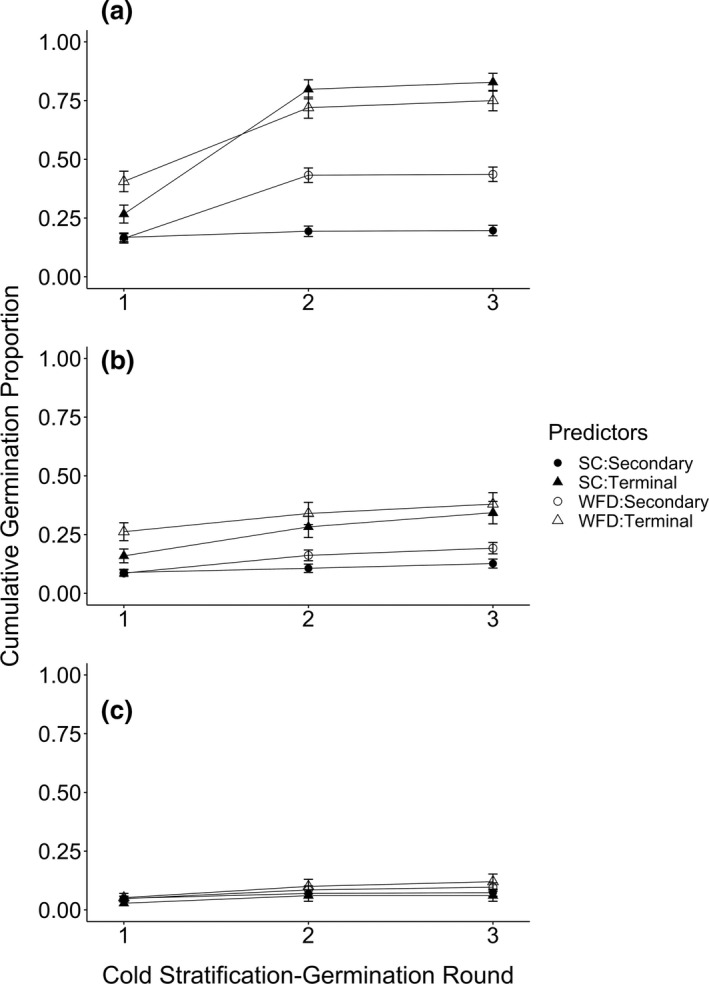
Cumulative *C. pitcheri* seed germination proportions over three cold stratification—germination rounds for each site‐capitulum position combination at temperature treatments (a) 20/10°C, (b) 25/10°C, and (c) 30/10°C (day/night). Values are fitted cumulative germination proportions and standard errors predicted by minimal adequate models for each cold stratification‐germination round and seeds collected in 2016

*Cirsium pitcheri* cumulative seed germination proportions were consistently higher for seeds from terminal capitula and at 20°C across all cold stratification‐germination rounds (Table 1, Figure 1). By the end of the third cold stratification‐germination round, seeds from secondary capitula at WFD had higher germination proportions than at SC across all temperatures (Table 1, Figure 1). However, cumulative germination proportions of all seeds at 25 and 30°C were more than 50% and 70% lower than those at 20°C, respectively, for both sites of origin and capitulum positions (Figure 1).

**TABLE 1:** *Cirsium pitcheri* cumulative germination proportions for each cold stratification‐germination round at temperature treatments 20/10, 25/10, and 30/10°C (day/night)

Predictor			Cold stratification‐germination^b^		
Site of origin^a^	Capitulum position	Temperature (day/night, °C)	Round one	Round two	Round three
SC	Terminal	20/10	0.267 ± 0.038	0.798 ± 0.040	0.828 ± 0.038
SC	Terminal	25/10	0.159 ± 0.029	0.283 ± 0.045	0.343 ± 0.048
SC	Terminal	30/10	0.028 ± 0.011	0.061 ± 0.024	0.061 ± 0.024
WFD	Terminal	20/10	0.406 ± 0.044	0.720 ± 0.045	0.750 ± 0.043
WFD	Terminal	25/10	0.262 ± 0.038	0.340 ± 0.047	0.380 ± 0.049
WFD	Terminal	30/10	0.052 ± 0.018	0.100 ± 0.030	0.120 ± 0.032
SC	Secondary	20/10	0.168 ± 0.019	0.194 ± 0.022	0.197 ± 0.022
SC	Secondary	25/10	0.089 ± 0.014	0.107 ± 0.018	0.127 ± 0.019
SC	Secondary	30/10	0.049 ± 0.010	0.070 ± 0.015	0.073 ± 0.015
WFD	Secondary	20/10	0.163 ± 0.020	0.432 ± 0.031	0.436 ± 0.031
WFD	Secondary	25/10	0.086 ± 0.014	0.162 ± 0.023	0.192 ± 0.024
WFD	Secondary	30/10	0.048 ± 0.010	0.085 ± 0.017	0.097 ± 0.018

Note

Seeds were collected in 2016 from terminal and secondary capitula at SC and WFD.

^a^ Sites of origin in Door County, Wisconsin abbreviated as Ship Canal Nature Preserve (SC) and Whitefish Dunes State Park (WFD).

^b^ Values are fitted cumulative germination proportions and standard errors for each cold stratification‐germination round.

Models that best fit the germination data differed across cold stratification‐germination rounds. In the first round, models included all three individual predictor effects (site of origin, capitulum position, temperature), as well as the site‐capitulum position (ANOVA, Deviance = 5.83, *p* = .02) and temperature‐capitulum position two‐way interactions (ANOVA, Deviance = 8.45, *p* = .01). Models for the second and third rounds, however, included the individual predictor effects, their three‐way interaction and all two‐way interactions (ANOVA, round two: Deviance = 9.66, round three: Deviance = 10.1, both *p* < .01).

### 3.2 Individual seed germination

**FIGURE 2:**
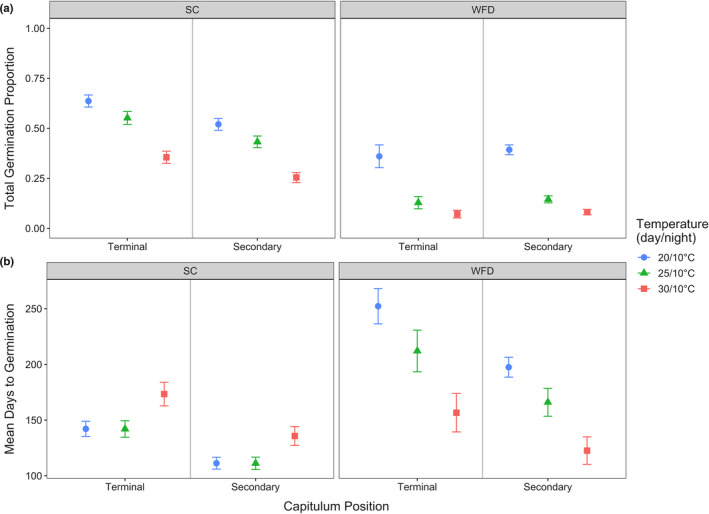
*Cirsium pitcheri* (a) total germination proportion and (b) mean days to germination for seeds from terminal and secondary capitulum positions at SC (left panel) and WFD (right panel). Values are (a) fitted total germination proportions and (b) day means with standard errors predicted by minimal adequate models at temperature treatments 20/10, 25/10, and 30/10°C (day/night) for seeds collected in 2017

When tracking the germination of individual seeds over time, seeds from SC had higher total germination proportions than WFD across all capitulum positions and temperatures (Table 2, Figure 2a). Overall, seeds from terminal capitula at SC had the highest total germination proportions which consecutively decreased at increasing temperature treatments (Figure 2a). For seeds from WFD, total germination proportions did not differ among capitulum positions, but also decreased at increasing temperature treatments (Figure 2a).

**TABLE 2:** *Cirsium pitcheri* total germination proportions and mean days to germination at temperature treatments 20/10, 25/10, and 30/10°C (day/night)

Predictors			Total germination proportion^b^	Days to germination^c^
Site of origin^a^	Capitulum position	Temperature (day/night, °C)		
SC	Terminal	20/10	0.636 ± 0.030	142 ± 6.80
SC	Terminal	25/10	0.552 ± 0.032	142 ± 7.40
SC	Terminal	30/10	0.355 ± 0.031	173 ± 10.6
WFD	Terminal	20/10	0.360 ± 0.057	252 ± 15.9
WFD	Terminal	25/10	0.128 ± 0.031	212 ± 18.7
WFD	Terminal	30/10	0.071 ± 0.019	157 ± 17.2
SC	Secondary	20/10	0.520 ± 0.030	111 ± 5.30
SC	Secondary	25/10	0.432 ± 0.029	111 ± 5.50
SC	Secondary	30/10	0.254 ± 0.025	136 ± 8.50
WFD	Secondary	20/10	0.393 ± 0.024	198 ± 8.90
WFD	Secondary	25/10	0.145 ± 0.017	166 ± 12.5
WFD	Secondary	30/10	0.081 ± 0.013	123 ± 12.3

Note

Seeds were collected in 2017 from terminal and secondary capitula at SC and WFD.

^a^ Sites of origin in Door County, Wisconsin abbreviated as Ship Canal Nature Preserve (SC) and Whitefish Dunes State Park (WFD).

^b^ Values are fitted total germination proportions and standard errors at the end of three cold stratification‐germination rounds.

^c^ Values are mean number of days to germination and standard errors across three cold stratification‐germination rounds.

At both sites of origin, seeds from terminal capitula took longer to germinate than those from secondary capitula across all temperature treatments except 30°C (Table 2, Figure 2b). For SC, seeds from terminal and secondary capitula had comparable mean times to germination at 20 and 25°C, but took longer to germinate at 30°C. The opposite was the case for seeds from WFD, for which seeds from terminal and secondary capitula took longer to germinate at 20°C, followed by 25°C, and then 30°C (Figure 2b).

### 3.3 Effect of seed weight on germination probability

**FIGURE 3:**
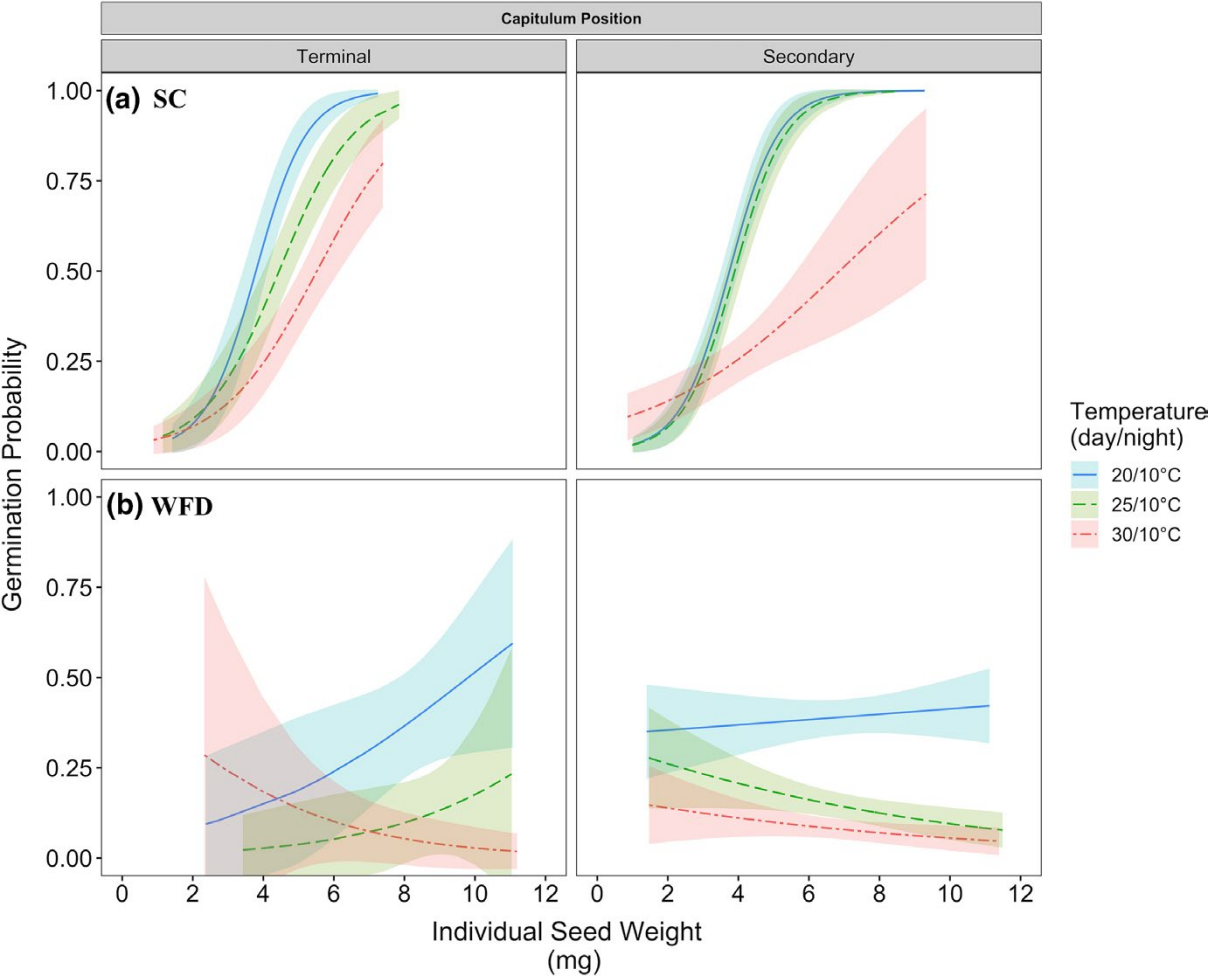
*Cirsium pitcheri* germination probability as a function of individual seed weight for seeds from terminal and secondary capitulum positions at (a) SC (top panel) and (b) WFD (bottom panel) in 2017. Lines are fitted regressions and 95% confidence intervals for *C. pitcheri* germination probabilities at temperature treatments 20/10, 25/10, and 30/10°C (day/night) predicted by minimal adequate models

Individual seed weight and its interactions with seed attribute predictors was a significant factor influencing the germination probability of individual seeds at the three temperature treatments (ANOVA, Deviance = 8.73, *p* = .012). At SC, the germination probability of individual seeds increased concurrently with seed weight across all temperature treatments regardless of capitulum position (Figure 3). However, at WFD, germination probabilities of individual seeds from terminal capitula at the three temperature treatments differed with increasing seed weight: they increased at 20 and 25°C and decreased at 30°C, but these effects were not significant (GLM, 20°C: *β* = .30, *p* = .10, 25°C: *β* = .34, *p* = .39, 30°C: *β* = −.34, *p* = .19). For seeds from secondary capitula at WFD, germination probabilities decreased with increasing seed weight at 25 and 30°C and remained relatively constant at 20°C (Figure 3).

### 3.4 Seed germination over time

**FIGURE 4:**
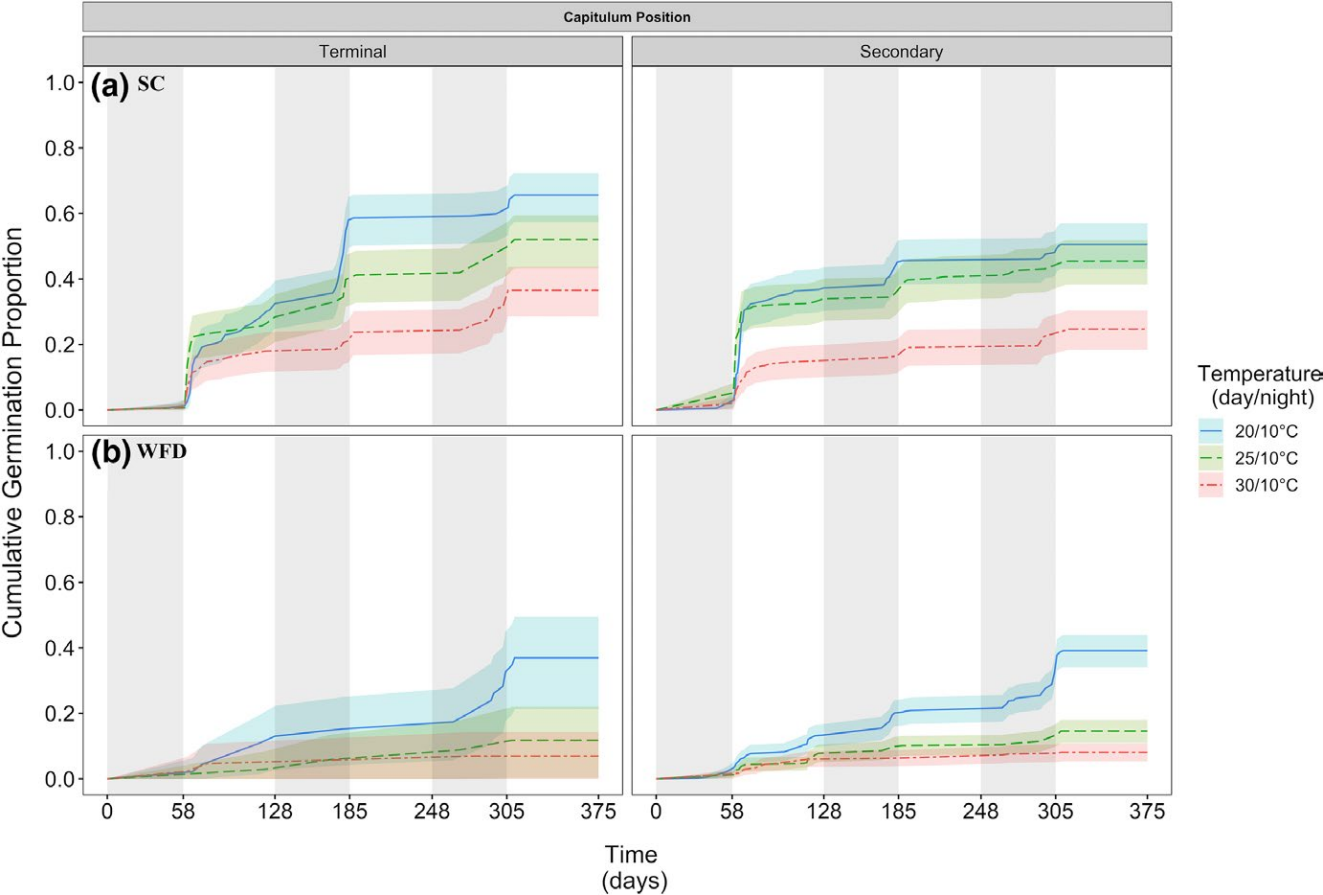
*Cirsium pitcheri* germination over time for seeds from terminal and secondary capitulum positions at (a) SC (top panel) and (b) WFD (bottom panel) in 2017. Lines are reverse survival curves (1 − Kaplan–Meier) and 95% confidence intervals for *C. pitcheri* germination at temperature treatments 20/10, 25/10, and 30/10°C (day/night). Periods of cold stratification at 3°C are shaded in gray

Survival curves describing the germination of individual seeds over time differed among temperature treatments and cold stratification‐germination rounds (Tables 3 and 4). Survival curves for seeds from terminal capitula at SC differed among all temperature treatments, while those from secondary capitula only differed among 20–30 and 25–30°C (Table 3, Figure 4). In contrast, survival curves for seeds from terminal capitula at WFD differed only among 20–25 and 20–30°C, while those from secondary capitula differed among all temperature treatments (Table 3). All survival curves for SC differed from those for WFD among all temperature treatments (Log‐rank test, Terminal: $\chi_{5}^{2}$ = 74.9, Secondary: $\chi_{5}^{2}$ = 237, both *p* < .001). Within the first cold stratification‐germination round, only survival curves for SC differed among temperature treatments (Table 4). In the second round, only survival curves for seeds from secondary capitula at SC differed among temperature treatments. Lastly, in the third cold stratification‐germination rounds, only survival curves for WFD differed among temperature treatments (Figure 4).

**TABLE 3:** Log‐rank test pairwise temperature comparisons of Kaplan–Meier survival curves for *Cirsium pitcheri* seeds from terminal and secondary capitula at SC and WFD in 2017

Site of origin^a^	Capitulum position	Temperature comparison (day/night, °C)^b^		
		20/10−25/10	25/10−30/10	20/10−30/10
SC	Terminal	4.70*	7.90**	27.4**
SC	Secondary	0.60	21.5***	30.6***
WFD	Terminal	6.2*	0.50	10.7**
WFD	Secondary	57.0***	7.50**	97.8***

^a^ Sites of origin in Door County, Wisconsin abbreviated as Ship Canal Nature Preserve (SC) and Whitefish Dunes State Park (WFD).

^b^ Values are chi‐squares on one degree of freedom ($\chi_{1}^{2}$).

* *p* < .05;

** *p* < .01;

*** *p* < .001.

**TABLE 4:** Log‐rank test comparisons of Kaplan–Meier survival curves by cold stratification‐germination round for *Cirsium pitcheri* seeds from terminal and secondary capitulum positions at SC and WFD in 2017

Site of origin^a^	Capitulum position	Cold stratification‐germination^b^		
		Round one	Round two	Round three
SC	Terminal	13.3**	5.00	0.00
SC	Secondary	9.50**	14.5***	0.40
WFD	Terminal	4.40	0.10	11.8**
WFD	Secondary	4.70	3.20	106**

^a^ Sites of origin in Door County, Wisconsin abbreviated as Ship Canal Nature Preserve (SC) and Whitefish Dunes State Park (WFD).

^b^ Values are chi‐squares on two degrees of freedom ($\chi_{2}^{2}$).

** *p* < .01;

*** *p* < .001.

### 3.5 Hazard ratio estimates for predictors

**FIGURE 5:**
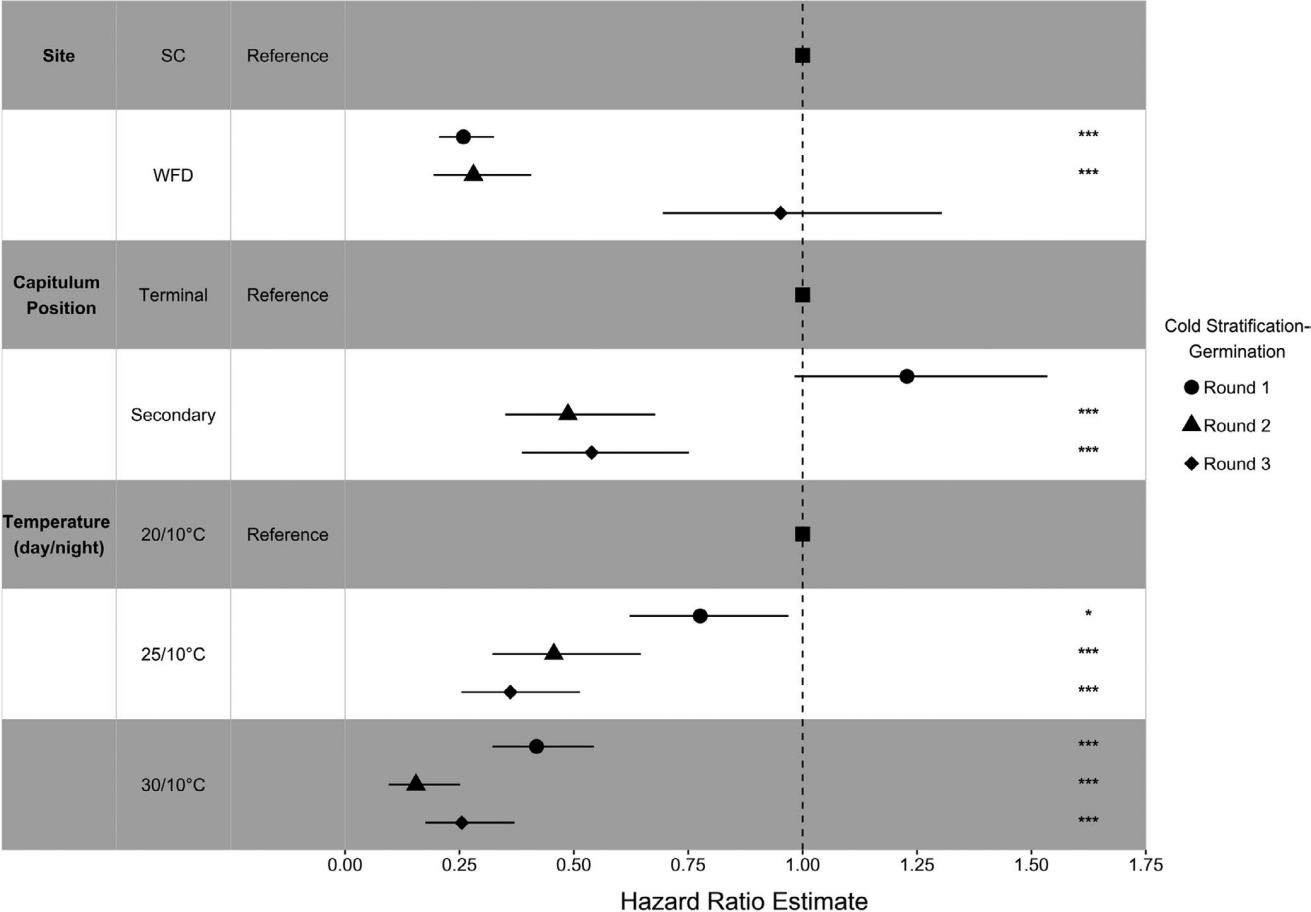
Hazard ratio estimates and 95% confidence intervals for predictor effects (site of origin, capitulum position, temperature) in Accelerated Failure Time (AFT) models at each cold stratification‐germination round. Stars denote predictor significance with respect to predictor reference level (*p < .05, ***p < .001)

Compared to seeds from SC, seeds from WFD were more than 70% less likely to germinate in the first and second cold stratification‐germination rounds, but as likely to germinate as those from SC in the third round (Figure 5). Seeds from secondary capitula were as likely to germinate as those from terminal capitula in the first round, but more than 45% less likely to germinate in the second and third rounds. Compared to 20°C, seeds at 25 and 30°C, respectively, were 22% and 58% less likely to germinate in the first round. In the second and third rounds, seeds were more than 50% and 70% less likely to germinate at 25 and 30°C, respectively (Figure 5).

## 4 DISCUSSION

Our study demonstrates that *C. pitcheri* is capable of producing phenotypically diverse cohorts of seeds, with seed attributes that influence the probability, timing and rate of germination of seeds at temperature treatments that simulate projected warming in the Great Lakes. Seed germination events were also broadly distributed over a 3‐year seedbank, which we simulated by subjecting seeds to three consecutive periods of cold stratification and warm‐temperature germination (cold stratification‐germination round).

### 4.1 Effect of capitulum position and site of origin on germination

In general, seeds from terminal capitula had higher germination proportions and took longer to germinate than those from secondary capitula at both sites of origin. Seeds from WFD also took longer to germinate than those from SC across all temperature treatments except 30°C. Previous studies have shown that a seed's developmental position on a parental plant can impact the length of seed dormancy and likelihood of germination (Baskin & Baskin, 2014; Lu et al., 2017). Our results indicate that site of origin and capitulum position is important seed attributes influencing patterns of germination in *C. pitcheri*.

The effects of seed weight on dormancy and germination, more specifically, have also been shown to depend on a seed's developmental position in a fruit or on a parental plant (Baskin & Baskin, 2014; Diggle, 1995; Susko & Lovett‐Doust, 2011). We had previously found that *C. pitcheri* seeds from terminal capitula are significantly heavier than those from secondary capitula and that flowering plants at both sites in 2017 produced fewer viable seeds in terminal and secondary capitula in 2016 (Gijsman et al., 2020). Following up on that work, here, we used seeds from senescing flowering plants in 2016 and 2017 to investigate the effects of *C. pitcheri* seed attributes on cumulative and individual seed germination, respectively. Hence, while we cannot make a direct comparison of our results for seeds collected in different years, it is likely that interannual variation in *C. pitcheri* seed production influenced seed attribute effects in our study. In addition, the position‐dependent effects on seed germination observed in this study likely reflect resource allocation strategies employed by maternal plants, resulting in the seed weight differences among capitulum positions seen in *C. pitcheri* plants at both sites.

### 4.2 Effect of seed weight on germination

Seed weight significantly influenced the germination probability of individual seeds at both sites, although to different extents. At SC, heavier seeds from both terminal and secondary capitula had higher germination probabilities than lighter seeds across all temperature treatments. In contrast at WFD, these effects were not as clear‐cut. For seeds from terminal capitula, germination probabilities increased with seed weight at 20 and 25°C and decreased at 30°C, but these effects were not significant. Given that terminal capitula at WFD produced few viable seeds in 2017 (Gijsman et al., 2020), our sample sizes for this capitulum position were small, which may have precluded our ability to distinguish any effects. For seeds from secondary capitula at WFD, seed weight significantly predicted germination probabilities at 25 and 30°C. At these high temperature treatments, heavier seeds were less likely to germinate than lighter seeds. These results are in accordance with previously published findings for *C. pitcheri* which indicate that seed weight influences seed dormancy and germination (Chen & Maun, 1999; Hamzé & Jolls, 2000).

Larger *C. pitcheri* seeds have also been reported to have higher chances of infection from seed‐ and soil‐borne pathogens (Chen & Maun, 1999). These results are especially noteworthy given that *L. planus* oviposits eggs at higher proportions in secondary capitula at WFD (Gijsman et al., 2020). When combined with the lower germination probability of heavier seeds produced in secondary capitula at WFD, our results highlight the potential impacts of *L. planus* on *C. pitcheri* that go beyond seed predation. In addition, fungal infection for seeds from WFD was a substantial issue in our study. While scarification of the seed coat by predating insects can aid with water imbibition and break seed dormancy (Han et al., 2018; Karban & Lowenberg, 1992), it can also impact seed quality, increase mold damage by disseminating fungal spores (Caneppele et al., 2003; Sinha, 1984), and impede seed germination (Dalgleish et al., 2012; Koptur, 2009; Tomaz et al., 2007). Although we did not assess the level of damage that seeds from WFD endured from predation by *L. planus*, our results suggest that it may impact the quality and dormancy of *C. pitcheri* seeds that survived predation. To better ascertain these potential effects, future studies should directly investigate the impact of *L. planus* on *C. pitcheri* seed germination and early life stages.

### 4.3 Effect of temperature on germination

Germination proportions for *C. pitcheri* seeds were highest at 20°C and decreased successively at the 25 and 30°C temperature treatments. The probability of seed germination at the higher temperature treatments also decreased over time. At 25°C, seed germination in the second cold stratification‐germination round was more than 50% less likely to occur than at 20°C, with that number increasing to 70% at 30°C. Such reductions in seed germination at 25 and 30°C suggest a critical temperature tolerance limit for *C. pitcheri* germination.

Survival curves describing the germination of seeds over time also indicate that germination rates differed at the different temperature treatments and across cold stratification‐germination rounds. For seeds from WFD, survival curves at the different temperature treatments only differed in the third round. In contrast, survival curves for seeds from terminal and secondary capitula at SC differed among temperature treatments only in the first and second rounds. These results highlight the considerably different physiologies that seeds from the two sites of origin and capitulum positions have and provide insight into the dormancy‐break patterns that *C. pitcheri* might experience under the predicted temperature rise in the Great Lakes region of North America.

While it is difficult to predict the extent to which the seed attribute effects in this study may impact the establishment and survival of *C. pitcheri* populations, our results suggest that climate change is a noteworthy threat that could increase the species' risk of extinction (Havens et al., 2012). Climate projections for the Great Lakes region predict annual temperatures to increase by 1.4 ± 0.6°C in the near‐term (2010–2039) (Hayhoe et al., 2010). Winter and spring precipitation is also projected to increase by 20% under a low emission scenario (Hayhoe et al., 2010). In conjunction with our results, predicted shifts in growing seasons and frequent extreme climate events suggest that climate change will significantly impact the timing and outcomes of seedling recruitment processes that are essential for the regeneration of *C. pitcheri* plant populations in dune ecosystems.

### 4.4 Implications for *C. pitcheri* populations

For *C. pitcheri*, variation in seed production, germination rate, and timing is likely an important bet‐hedging strategy that ensures diversity in the timing of population establishment events in a heterogeneous environment (Giesel, 2003; Sales et al., 2013) and under changing environmental conditions (Lu et al., 2017). In addition, because flowering anthesis is asynchronous in *C. pitcheri*, differences in the floral phenology between terminal and secondary capitula further contribute to the temporal variation in population establishment events. Terminal capitula in *C. pitcheri* develop, mature, and set seeds that are also heavier, earlier than secondary capitula (Gijsman et al., 2020; USFWS, 2002). These differences in floral phenology and seed production between capitulum positions therefore may result in contrasting seed performances for *C. pitcheri* populations, with weight‐dependent trade‐offs in dispersal, burial, dormancy, germination, and seedling emergence and establishment (de Ruiz, 2002; Lu et al., 2017; Susko & Lovett‐Doust, 2000; Turnbull et al., 2004; Venable & Brown, 1988). For instance, *C. pitcheri* seed germination and seedling emergence have been found to be negatively correlated with seed burial depth (Chen & Maun, 1999; Hamzé & Jolls, 2000). Yet, seedlings emerging from larger seeds produce longer roots that enhance their establishment in the dunes (Chen & Maun, 1999). Hence, while seeds from terminal capitula develop earlier, are heavier, have longer dormancy periods and are more likely to germinate than those from secondary capitula, as we have demonstrated, they may not be dispersed as far, may be buried at different depths and, if still viable, produce more robust seedlings due to their larger size. Altogether, these weight‐dependent trade‐offs in seed germination ecology and variation in phenology may contribute to population persistence in disturbance‐prone dune environments.

Similar strategies and germination patterns have been reported for plant species with two flowering‐fruiting events. For example, seed production in *Centaurea eriophora* (Asteraceae) depends on the size and flowering period of capitula (de Ruiz, 2002). Seeds from small capitula, which flower later than large capitula, are lighter and initially produce seedlings that are less robust (de Ruiz, 2002). Likewise, Gurvich et al. (2004) found that seeds from early‐flowering *Bidens pilosa* (Asteraceae) plants (referred to as early type) are heavier, have faster germination rates and lower germination probabilities than those from normal‐type plants. In both cases, the prolongation of flowering and fruiting periods resulted in the production of seeds with variable traits that are essential for the initial establishment of populations in the face of environmental change (de Ruiz, 2002; Gurvich et al., 2004). Nonetheless, it is important to note that while such reproductive strategies can produce a wide array of seeds, their success is also highly dependent on site dynamics and climatic conditions such as temperature, the latter which our study indicates has strong effects on seed germination.

## 5 CONCLUSION

In this study, we found that *C. pitcheri* produces seeds with germination events that are widely distributed over a 3‐year soil seed bank and with seed attributes that significantly influence the timing and probability of germination under projected warming. Our results also indicate that terminal capitula produce seeds with the highest germination probabilities and that high temperatures drastically impact *C. pitcheri* germination. With climate change posing a significant threat to *C. pitcheri* populations, actions that increase the likelihood of germination and seedling survival, such as selectively using larger seeds or seeds sourced from terminal capitula, may help ensure population persistence and success in conservation and restoration efforts.

## CONFLICT OF INTEREST

The authors declare that they have no conflicts of interest.

## AUTHOR CONTRIBUTION

**Finote Gijsman:** Conceptualization (equal); Data curation (lead); Formal analysis (lead); Funding acquisition (lead); Investigation (equal); Methodology (equal); Writing‐original draft (lead); Writing‐review & editing (equal). **Pati Vitt:** Conceptualization (equal); Formal analysis (supporting); Funding acquisition (supporting); Investigation (equal); Methodology (equal); Project administration (lead); Supervision (lead); Writing‐review & editing (equal).

## Supporting information

Table S1

## Data Availability

Data in this study are available on Dryad: https://doi.org/10.5061/dryad.xksn02vdw.
