# Supplementary material for: Seed size and capitulum position drive germination and dormancy responses to projected warming for the threatened dune endemic Cirsium pitcheri (Asteraceae)
Source: Ecol Evol. 2020 Dec 21;11(2):955–66. doi: 10.1002/ece3.7109 (PMC7820163; doi:10.1002/ece3.7109)
Supplement: Supplementary file 1 — Table S1 [file ECE3-11-955-s001.docx]

**Table S1.** *Cirsium pitcheri* seed sample sizes for germination trials at temperature treatments (20/10°C, 25/10°C and 30/10°C, day/night) using seeds from terminal and secondary capitula collected at SC and WFD in 2016 and 2017

| **Seed Collection Year^a^** | **Site of Origin^b^** | **Capitulum Position** | **Temperature**  **(day/night, °C)** | **Seed Sample Size** | **Number of Replicates** |
| --- | --- | --- | --- | --- | --- |
| 2016 | SC | Terminal | 20/10 | 99 | 5 |
| 2016 | SC | Terminal | 25/10 | 99 | 5 |
| 2016 | SC | Terminal | 30/10 | 99 | 5 |
| 2016 | SC | Secondary | 20/10 | 320 | 16 |
| 2016 | SC | Secondary | 25/10 | 300 | 15 |
| 2016 | SC | Secondary | 30/10 | 300 | 15 |
| 2016 | WFD | Terminal | 20/10 | 100 | 5 |
| 2016 | WFD | Terminal | 25/10 | 100 | 5 |
| 2016 | WFD | Terminal | 30/10 | 100 | 5 |
| 2016 | WFD | Secondary | 20/10 | 259 | 13 |
| 2016 | WFD | Secondary | 25/10 | 260 | 13 |
| 2016 | WFD | Secondary | 30/10 | 259 | 13 |
| 2017 | SC | Terminal | 20/10 | 157 | 10 |
| 2017 | SC | Terminal | 25/10 | 148 | 10 |
| 2017 | SC | Terminal | 30/10 | 156 | 10 |
| 2017 | SC | Secondary | 20/10 | 204 | 14 |
| 2017 | SC | Secondary | 25/10 | 209 | 14 |
| 2017 | SC | Secondary | 30/10 | 199 | 13 |
| 2017 | WFD | Terminal | 20/10 | 46 | 3 |
| 2017 | WFD | Terminal | 25/10 | 34 | 3 |
| 2017 | WFD | Terminal | 30/10 | 43 | 3 |
| 2017 | WFD | Secondary | 20/10 | 388 | 26 |
| 2017 | WFD | Secondary | 25/10 | 384 | 26 |
| 2017 | WFD | Secondary | 30/10 | 382 | 25 |

^a^Seeds from 2016 were used in germination trials tracking cumulative seed germination while those from 2017 were used in trials tracking individual seed germination

^b^Sites of origin in Door County, Wisconsin abbreviated as Ship Canal Nature Preserve (SC) and Whitefish Dunes State Park (WFD)
